# The age-related failure of adaptive responses to contractile activity in skeletal muscle is mimicked in young mice by deletion of Cu,Zn superoxide dismutase

**DOI:** 10.1111/j.1474-9726.2010.00635.x

**Published:** 2010-12

**Authors:** Aphrodite Vasilaki, Jack H van der Meulen, Lisa Larkin, Dawn C Harrison, Timothy Pearson, Holly Van Remmen, Arlan Richardson, Susan V Brooks, Malcolm J Jackson, Anne McArdle

**Affiliations:** 1 School of Clinical Sciences, University of LiverpoolLiverpool L69 3GA, UK; 2 Institute of Gerontology, University of MichiganAnn Arbor, MI, USA; 3 University of Texas Health Science CenterSan Antonio, TX, USA

**Keywords:** aging, AP-1, exercise, NFκB, sarcopenia, skeletal muscle

## Abstract

In muscle, aging is associated with a failure of adaptive responses to contractile activity, and this is hypothesized to play an important role in age-related loss of muscle mass and function. Mice lacking the Cu,Zn superoxide dismutase (Cu,ZnSOD, SOD1) show an accelerated, age-related loss of muscle mass and function. This work determined whether adult mice lacking Cu,ZnSOD (*Sod1*^*−/−*^ mice) show a premature failure of adaptive responses to contractions in a similar manner to old wild-type (WT) mice. Adult *Sod1*^*−/−*^ mice (6–8 months of age) had a ∼ 30% reduction in *gastrocnemius* muscle mass compared with age-matched WT mice. This lower muscle mass was associated with an activation of DNA binding by NFκB and AP-1 at rest. Measurements of the activity of reactive oxygen species (ROS) in single fibres from the muscles of *Sod1*^*−/−*^ mice at rest indicated an elevation in activity compared with fibres from WT mice. Following 15 min of isometric contractions, muscle fibres from WT mice showed an increase in the intracellular ROS activities and activation of NFκB and AP-1, but no changes in either ROS activity or NFκB and AP-1 activation were seen in the muscles of *Sod1*^*−/−*^ mice following contractions. This pattern of changes mimics that seen in the muscles of old WT mice, suggesting that the attenuated responses to contractile activity seen in old mice result from chronic exposure to increased oxidant activity. Data support the use of the *Sod1*^*−/−*^ mouse model to evaluate potential mechanisms that contribute to the loss of muscle mass and function in the elderly.

## Introduction

Age-related loss of muscle mass and strength is the major contributor to frailty and loss of independence in the elderly (Marcell, 2003) such that by the age of 70, the cross-sectional area of skeletal muscle is reduced by 25–30% and muscle strength is reduced by 30–40% (Porter *et al.*, 1995). Increased levels of products of oxidative reactions are associated with the age-related decline in muscle mass and function. Skeletal muscle of aged rodents contains the increased amounts of the products of oxidative damage to biomolecules such as lipid, DNA and proteins in comparison with young or adult rodents (Nohl, 1993; Lass *et al.*, 1998; Schoneich, 1999; Reid & Durham, 2002; Drew *et al.*, 2003; Sastre *et al.*, 2003; Broome *et al.*, 2006; Vasilaki *et al.*, 2007) although definitive evidence for a role of oxidative damage in the development of age-related loss of muscle mass and function is lacking (Pérez *et al.*, 2009).

Tissue activities of reactive oxygen species (ROS) are regulated by protective enzymatic systems. The key enzymatic antioxidants appear to be the superoxide dismutases, catalase and glutathione peroxidases with major antioxidant roles also proposed to be undertaken by thiol peptides (e.g. glutathione) and proteins such as thioredoxin and the peroxiredoxins (Halliwell & Gutteridge, 2007). The superoxide dismutases have different locations in the cell: CuZnSOD (SOD1) is primarily cytosolic, although a proportion of the cellular CuZnSOD is also found in the mitochondrial intermembrane space (Okado-Matsumoto & Fridovich, 2001), MnSOD (SOD2) is found within mitochondria and extracellular SOD (SOD3) is found within the extracellular space.

The generation of ROS in skeletal muscle increases during contraction (Balon & Nadler, 1994; O’Neill *et al.*, 1996; McArdle *et al.*, 2001), and ROS interact with multiple cell signalling and regulatory pathways to modulate the changes in gene expression in a number of cell types (Jackson *et al.*, 2002). The initial adaptive responses that occur in skeletal muscle following an increase in ROS activity induced by isometric contractions are in the protective enzymatic systems and in the stress or heat shock proteins (HSPs), and increased muscle content of several of these proteins has been associated with significant protection against subsequent cellular damage (Hollander *et al.*, 2003; McArdle *et al.*, 2004a, b). Contraction-induced changes in ROS modulate some of the adaptive responses that occur in skeletal muscle following contractile activity. A single isometric contraction protocol in mouse muscle, previously shown to increase muscle ROS generation, increased the activity of muscle antioxidant defence enzymes such as superoxide dismutase (SOD) and catalase together with HSP60 and HSP70 content (McArdle *et al.*, 2001), changes that were replicated in the studies of human muscle (Khassaf *et al.*, 2001). Presupplementation with vitamin C (Khassaf *et al.*, 2003) or other antioxidants (Jackson *et al.*, 2004) reduced these adaptive responses, supporting the possibility that these adaptations were regulated by ROS. Complimentary data from Gomez-Cabrera *et al.* (2008) indicate that treatment of rats with antioxidants prevented several exercise-induced changes in skeletal muscle gene expression.

Many transcriptional responses of tissues to changes in ROS involve the activation of redox-sensitive transcription factors (Jackson *et al.*, 2002). Two such factors, nuclear factor κB (NFκB) and activator protein-1 (AP1), are involved in the upregulation of antioxidant enzymes such as SOD and catalase in response to oxidative stress (Zhou *et al.*, 2001; Jackson *et al.*, 2002). Expression of heat shock proteins in response to acute stress in eukaryotic cells is primarily regulated by the transcription factor, heat shock factor 1 (HSF1; Cotto & Morimoto, 1999). NFκB, AP-1 and HSF transcription factors were activated in the skeletal muscle of adult mice following an isometric contraction protocol that also increased the ROS generation and increased the content of HSPs, SOD and catalase (Vasilaki *et al.*, 2006c).

Cells and tissues from old mammals demonstrate a chronic activation of redox-responsive transcription factors such as NFκB and AP1, and this is associated with an attenuated ability of cells and tissues from old mammals to respond to a variety of stresses by further activation of these transcription factors and an increase in the activity of antioxidant defence enzymes and content of HSPs (Locke & Tanguay, 1996; Muramatsu *et al.*, 1996; Rao *et al.*, 1999; Heydari *et al.*, 2000; Njemini *et al.*, 2002; Demirel *et al.*, 2003). The increase in antioxidant defence enzyme activity and HSP content evident in the muscles of adult rodents following isometric contractions were also abolished in the muscles of old rodents (Vasilaki *et al.*, 2002, 2006c), and this inability to adapt appeared to be because of the lack of complete activation of the appropriate transcription factors (Vasilaki *et al.*, 2006c). This age-related inability to adapt by changes in gene expression plays a critical role in the development of functional deficits that occur with aging in skeletal muscle. Studies using transgenic mice overexpressing HSP70 in skeletal muscle demonstrated that increased muscle content of this protein provided protection against the fall in specific force associated with aging and facilitated rapid and successful regeneration following contraction-induced damage in the muscles of old mice compared with the impaired regeneration and recovery normally observed in old wild-type (WT) mice (McArdle *et al.*, 2004a). This protection was associated with maintenance of the ability of muscles of old HSP70 overexpressor mice to activate NFκB following isometric contractions (Broome *et al.*, 2006). Thus, the capacity of skeletal muscle cells to respond to acute increases in ROS activity and maintain cellular ROS homoeostasis appears limited and may be inadequate when muscle is subjected to chronic increased ROS activity such as in the muscles of old rodents.

The hypothesis that an increased generation of oxidants *in vivo* plays a key role in age-related tissue dysfunction has been examined with inconsistent results in nonmammalian systems using overexpression of either Cu,ZnSOD, catalase or both in *drosophila* (e.g. see Orr & Sohal, 1993, 1994; Orr *et al.*, 2003) and treatment of *Caenorhabditis elegans* with a MnSOD and catalase mimetic (Melov *et al.*, 2000). Studies in which protective enzymes were overexpressed in mice have also provided contradictory data; mice overexpressing catalase in mitochondria had a significant increase in lifespan (Schriner *et al.*, 2005), whereas mice overexpressing Cu,ZnSOD showed no such increase (Huang *et al.*, 2000). Previous studies have indicated that mice lacking Cu,ZnSOD (*Sod1*^*−/−*^ mice) show an accelerated age-related loss of skeletal muscle mass and function (Muller *et al.*, 2006) and potentially provide a useful model to study the role of a chronic oxidative stress in the loss of muscle mass and function (Jackson, 2006).

The first aim of the current study was to determine whether a chronic increase in cellular oxidative stress in quiescent muscles of adult *Sod1*^*−/−*^ mice leads to the activation of redox-regulated adaptive responses in a similar manner to that seen in quiescent muscle of old WT mice. Secondly, we aimed to determine whether the muscles of adult *Sod1*^*−/−*^ mice fail to further activate adaptive responses following an isometric contraction protocol mimicking the failure seen in the muscle of old WT mice.

Our hypothesis was that the skeletal muscles of adult *Sod1*^*−/−*^ mice are exposed to a chronic increase in oxidant activity in a similar manner to the muscles of old WT mice and that this increase in oxidative stress results in the modification of redox-responsive transcription factors at rest and in response to physiological processes such as nondamaging contractile activity in a similar manner to that seen during aging.

## Results

**Fig. 1:**
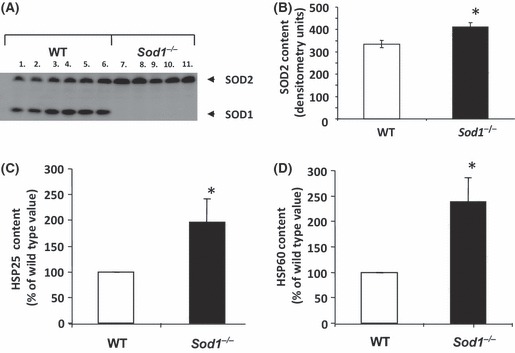
(A) Western blot analyses of Cu,ZnSOD (SOD1) and MnSOD (SOD2) in *gastrocnemius* muscles from adult wild-type (WT) (lanes 1–6) and *Sod1*^*−/−*^ (lanes 7–11) mice. (B) MnSOD (SOD2) contents of *gastrocnemius* muscles obtained by quantification of western blots using densitometry are shown as mean ± SEM. There was a significant increase in MnSOD content in the muscle of *Sod1*^*−/−*^ mice in comparison with that from WT mice, (*P < 0.05 compared with values from muscle of WT mice). (C) Quantification of western blot analyses of HSP25 in *gastrocnemius* muscles from adult WT and *Sod1*^*−/−*^ mice. These data are shown as mean ± SEM. There was a significant increase in the content of HSP25 in the muscle of *Sod1*^*−/−*^ mice in comparison with that from WT mice, (*P < 0.05 compared with values from muscle of WT mice). (D) Quantification of western blot analyses of HSP60 in *gastrocnemius* muscles from adult WT and *Sod1*^*−/−*^ mice. These data are shown as mean ± SEM. There was a significant increase in the content of HSP60 in the muscle of *Sod1*^*−/−*^ mice in comparison with that from WT mice, (*P < 0.05 compared with values from muscle of WT mice).

Western blots of the Cu,ZnSOD (SOD1) protein in *gastrocnemius* muscles from adult WT and *Sod1*^*−/−*^ mice are shown in Fig. 1A together with representative blots for MnSOD (SOD2). Muscles from the *Sod1*^*−/−*^ mice had no detectable Cu,ZnSOD protein. In contrast, muscles from the *Sod1*^*−/−*^ mice showed a small but statistically significant increase in MnSOD content (Fig. 1B).

*Gastrocnemius* muscles from the adult *Sod1*^*−/−*^ mice had a reduced mass compared with those from the age-matched, adult WT mice which remained reduced when body weight was accounted for (Table 1). This was associated with an increase in the content of two heat shock proteins (HSP25 and HSP60), (Fig. 1C, D).

**Table 1:** Gastrocnemius and body weight of *Sod1*^*−/−*^ and age-matched wild-type (WT) control mice. *P < 0.05 cf. WT

	WT	*Sod1*^*−/−*^
Gastroc wt (mg)	163 (6.4)	113.9 (2.1)*
Body wt (g)	31.3 (1.5)	27.7 (0.8)
Gastroc:Body wt	5.25 (0.16)	4.1 (0.1)*

### ROS activities in the quiescent muscles of WT and *Sod1*^*−/−*^ mice

**Fig. 2:**
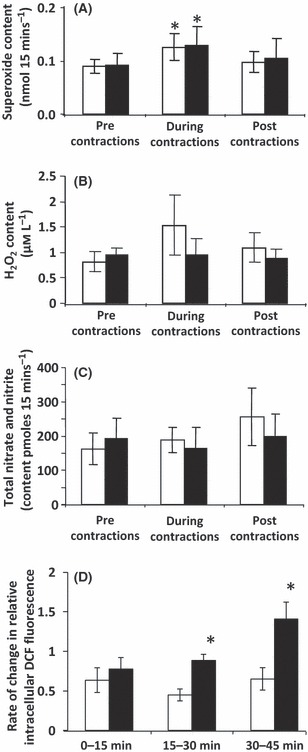
(A) Reduction of cytochrome *c* (expressed as superoxide equivalents) in microdialysates from the *gastrocnemius* muscles from wild-type (WT) (□) and *Sod1*^*−/−*^ mice (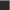) mice. Muscles underwent a 15-min period of demanding contractile activity during the period shown. Data are shown as the mean ± SEM. *P < 0.05 compared with microdialysates from the same group of mice. (B) Hydrogen peroxide content of microdialysates from the *gastrocnemius* muscles from WT (□) and *Sod1*^*−/−*^ mice (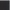) mice. Muscles underwent 15-min periods of demanding contractile activity during the period shown. Data are shown as the mean ± SEM. (C) Total nitrate and nitrite content of microdialysates from the *gastrocnemius* muscles from WT (□) and *Sod1*^*−/−*^ mice (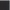) mice. Muscles underwent 15-min periods of demanding contractile activity during the period shown. Data are shown as the mean ± SEM. (D) CM-DCF fluorescence from single mature skeletal muscle fibres of the *flexor digitorum brevis* muscles of WT (□) and *Sod1*^*−/−*^ mice (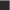) mice at rest. Values are shown for three 15-min periods and presented as the rate of change of fluorescence normalized to the initial fluorescence measurements as previously described (Palomero *et al.*, 2008). Data are shown as the mean ± SEM. A significant main effect of strain was observed. *P < 0.05 compared with values from WT mice at the same time point.

Two different approaches were used to examine the activities of ROS in muscle from the *Sod1*^*−/−*^ and WT mice. A general measure of intracellular ROS activities was obtained by monitoring the oxidation of dichlorodihydrofluorescein (DCFH) in isolated intact fibres from the *flexor digitorum brevis* (FDB) muscle of the mice, and the activities of superoxide, nitric oxide and hydrogen peroxide in the *gastrocnemius* muscle extracellular space were monitored using microdialysis techniques. No differences in the reduction of cytochrome *c*, nitrate and nitrite contents (indicating NO release) or hydrogen peroxide content of the microdialysates were seen in quiescent muscles of *Sod1*^*−/−*^ and WT mice (Fig. 2A–C). In contrast, the oxidation of DCFH was significantly greater in quiescent fibres from the FDB of *Sod1*^*−/−*^ mice compared with quiescent fibres from WT mice (Fig. 2D).

### Activation of NFκB and AP-1 in the quiescent muscles of WT and *Sod1*^*−/−*^ mice

**Fig. 3:**
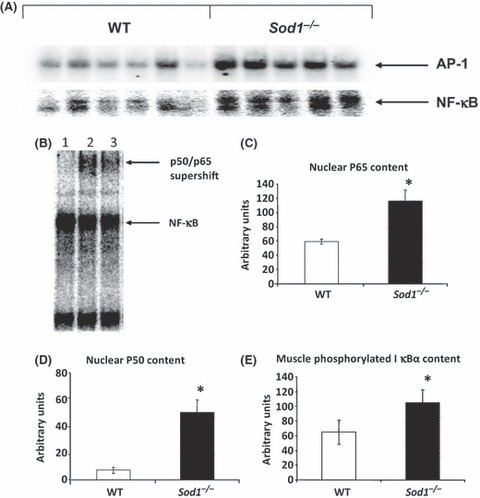
(A) NFkB and AP-1 DNA binding activity of nuclear extracts from quiescent *gastrocnemius* muscles of wild-type (WT) and *Sod1*^*−/−*^ mice. (B) Supershift analysis of the electrophoretic mobility shift assays for *gastrocnemius* muscles of *Sod1*^*−/−*^ mice showing the presence of P50 (lane 2) and P65 (lane 3) in comparison with no antibody additions (lane 1). (C) Quantification of western blots to show the relative content of P65 in nuclear extracts from quiescent *gastrocnemius* muscles of WT and *Sod1*^*−/−*^ mice. Data are arbitrary units obtained from densitometry and are presented as the mean ± SEM. *P < 0.05 compared with values from muscle of WT mice. (D) Quantifications of western blots to show the relative content of P50 in nuclear extracts from quiescent *gastrocnemius* muscles of WT and *Sod1*^*−/−*^ mice. Data are arbitrary units from densitometry and are presented as the mean ± SEM. *P < 0.05 compared with values from muscle of WT mice. (E) Quantification of western blots to show the content of phosphorylated IκBα in the quiescent *gastrocnemius* muscles of WT and *Sod1*^*−/−*^ mice.*P < 0.05 compared with values from muscle of WT mice.

**Fig. 4:**
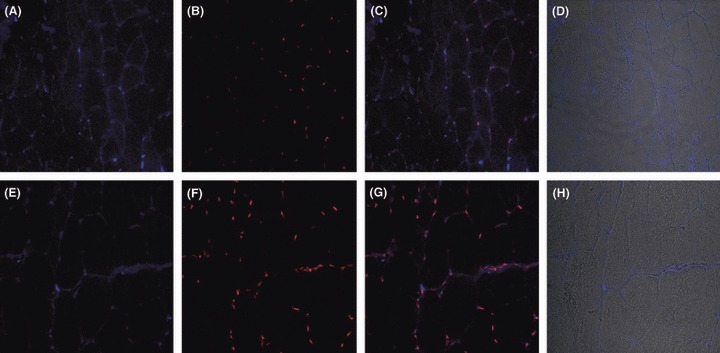
Transverse sections of quiescent *gastrocnemius* muscle of wild-type (A–D) and *Sod1*^*−/−*^ (E–H) mice stained for p65 (blue, A and E), for nuclei with propidium iodide (red, B and F), an overlay of propidium iodide and p65 (C and G) and an overlay of light microscope image with p65 (D and H). Sections stained with secondary antibody alone were negative (data not shown).

**Fig. 5:**
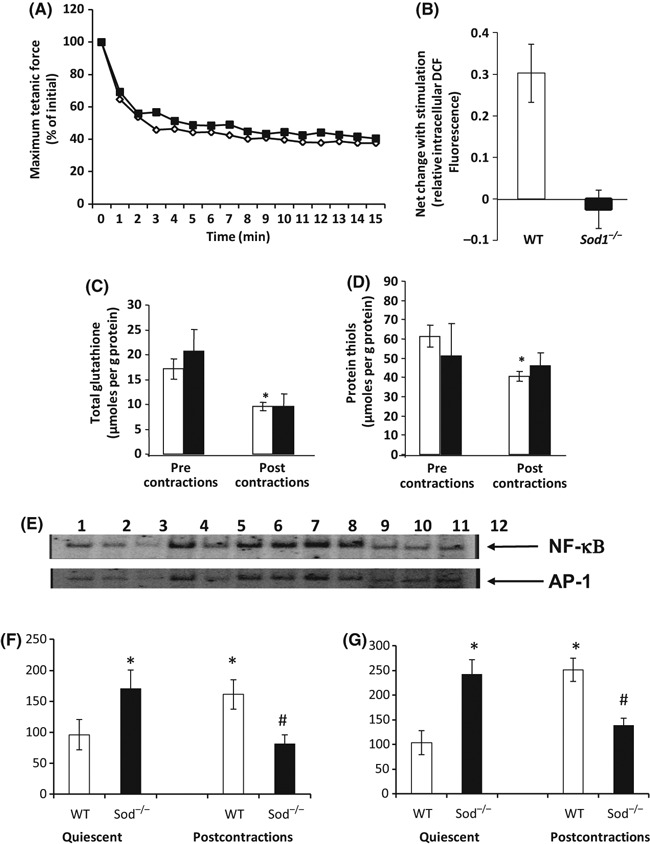
(A) Relative change in maximum forces generated by the *gastrocnemius* muscles of wild-type (WT) (◊) and *Sod1*^*−/−*^ (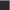) mice during the contraction protocol. Values are expressed as a percentage of the maximum force generated by that group of mice at time 0. (B) Net change on CM-DCF fluorescence from single mature skeletal muscle fibres of the *flexor digitorum brevis* muscles of WT (□) and *Sod1*^*−/−*^ mice (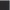) mice following 15-min contractile activity. Values are presented as the rate of change of fluorescence normalized to the initial fluorescence measurements as previously described (Palomero *et al.*, 2008). Data are shown as the mean ± SEM. (C) Total glutathione content of *gastrocnemius* muscles from adult WT (□) and *Sod1*^*−/−*^ mice (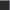) prior to contractile activity and at 15 min postcontractions. Data are shown as the mean ± SEM. *P < 0.05 compared with values from muscle of WT mice. (D) Total protein thiol content of *gastrocnemius* muscles from adult WT (□) and *Sod1*^*−/−*^ mice (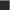) prior to contractile activity and at 15 min postcontractions. Data are shown as the mean ± SEM. *P < 0.05 compared with values from muscle of WT mice. (E) Representative electrophoretic mobility shift assays (EMSAs) showing NFκB and AP-1 binding activity of nuclear extracts from *gastrocnemius* muscles of WT (lanes 1–3) and *Sod1*^*−/−*^ mice (lanes 4–6) prior to contractile activity and of WT (lanes 7–9) and *Sod1*^*−/−*^ mice (lanes 10–12) at 15 min postcontractions. (F) Quantification of EMSA NFκB binding activity of nuclear extracts from gastrocnemius muscles of five WT and five *Sod1*^*−/−*^ mice. *P < 0.05 cf quiescent WT muscles; #*P* < 0.05 cf. Quiescent *Sod1*^*−/−*^ muscles. (G) Quantification of EMSA AP-1 binding activity of nuclear extracts from gastrocnemius muscles of five WT and five *Sod1*^*−/−*^ mice. *P < 0.05 cf quiescent WT muscles; #*P* < 0.05 cf. Quiescent *Sod1*^*−/−*^ muscles.

Electrophoretic mobility shift assays (EMSA) of nuclear extracts from the quiescent muscles of adult *Sod1*^*−/−*^ mice and adult WT mice are shown in Fig. 3A. These data show a 70% and 142% increase in DNA binding activity of NFκB and AP-1, respectively, in the nuclear extracts of muscles from *Sod1*^*−/−*^ compared with those from age-matched WT mice (Fig. 5F, G). Detailed analyses of the subunit composition of the activated NFκB complex seen in muscles from *Sod1*^*−/−*^ mice are shown in Fig. 3B. Addition of an antibody to either p50 or p65 to the nuclear extract from muscle of the *Sod1*^*−/−*^ mice resulted in the production of a supershift band, suggesting that these two proteins are present in the activated complex. Quantification of western blots for p65 and p50 proteins in nuclear extracts is shown in Fig. 3C, D and indicates a significant increase in both proteins in the nuclear extracts from muscle *Sod1*^*−/−*^ mice compared with those from WT mice. As a further measure of activation of NFκB, the muscle content of phosphorylated IκBα was examined and this was also found to be elevated in the muscle of *Sod1*^*−/−*^ mice. Figure 4 demonstrates the presence of p65 in the myonuclei of WT and *Sod1*^*−/−*^ mice.

### Effect of isometric contractions on tetanic force generation, ROS activities, activation of NFκB and AP-1 and thiol content in the muscles of WT and *Sod1*^*−/−*^ mice

The maximum tetanic force generation by the gastrocnemius muscles was significantly reduced in the muscles of *Sod1*^*−/−*^ mice compared with WT mice (*Sod1*^*−/−*^ mice: 2140 ± 161 mN vs. WT mice: 3770 ± 315 mN). The effects of the isometric contraction protocol on force generation are shown in Fig. 5A. The contraction protocol resulted in a similar percentage loss of force generation with no evidence of altered rates of fatigue between the muscles of *Sod1*^*−/−*^ and WT mice. The effects of contractile activity on reduction of cytochrome *c* (used as an index of superoxide activity), nitrate and nitrite contents (indicating NO release) and hydrogen peroxide content of the microdialysates are shown in Fig. 2. Contractile activity increased significantly the reduction of cytochrome *c* in microdialysates from the muscles of both *Sod1*^*−/−*^ and WT mice, but had no significant effect on the NO or hydrogen peroxide content of microdialysates from the muscles of either group of mice. The mean hydrogen peroxide content of microdialysates from the muscles of the *Sod1*^*−/−*^ mice during contractions was approximately 50% greater than the quiescent value, but the change in content was not statistically significant.

The total glutathione content and total protein thiol content of muscles from adult *Sod1*^*−/−*^ and WT mice prior to and immediately after the contraction protocol are shown in Fig. 5C, D. Both groups showed a tendency for a fall in the muscle total thiol and glutathione contents following the contractions, and this was statistically significant for the changes in WT mice, but there were no significant differences between the values for WT and *Sod1*^*−/−*^ mice.

Following the isometric contraction protocol, muscles of adult WT mice showed a significant increase in the DNA binding activity of NFκB and AP-1 (61% and 150% respectively, Fig. 5E–G). In contrast, there was a significant fall in the DNA binding activity of NFκB and AP-1 in the muscles of adult *Sod1*^*−/−*^ mice following isometric contractions (Fig. 5E–G).

### Effect of contractile activity on ROS activities in fibres isolated from muscles of WT and *Sod1*^*−/−*^ mice

Isolated fibres from the *flexor digitorum longus* muscles of the WT *Sod1*^*−/−*^ mice were subject to 15 min of contractile activity in culture as previously described (Palomero *et al.*, 2008). Fibres from WT mice showed a significant increase in DCF formation during the contractions, but no increase was seen from the fibres of *Sod1*^*−/−*^ mice (Fig. 5B).

## Discussion

Although it is well documented that tissues, such as skeletal muscle, from aging organisms contain increased amounts of the products of oxidative reactions (Nohl, 1993; Lass *et al.*, 1998; Schoneich, 1999; Reid & Durham, 2002; Drew *et al.*, 2003; Sastre *et al.*, 2003; Broome *et al.*, 2006; Vasilaki *et al.*, 2007), whether oxidative damage plays a direct role in fundamental processes of aging remains unclear (Muller *et al.*, 2007b; Pérez *et al.*, 2009). In recent years, it has become widely recognized that ROS are not inevitably damaging. Moreover, ROS exert physiological roles in cell signalling processes, and cells adapt to increases in the generation or activity of ROS by increasing the expression of proteins that protect against oxidative damage (McArdle *et al.*, 2001; Jackson *et al.*, 2002). A key process by which this occurs is through the activation of redox-sensitive transcription factors (Jackson *et al.*, 2002). NFκB and AP1 are involved in the upregulation of antioxidant enzymes such as SOD and catalase in response to oxidative stress (Zhou *et al.*, 2001; Jackson *et al.*, 2002), and HSF1 is important for HSP expression in response to acute stress (Cotto & Morimoto, 1999). In adult mice, activation of NFκB, AP-1 and HSF occurs in skeletal muscle following an isometric contraction protocol that also caused increased ROS generation and resulted in an increased muscle content of HSPs, SOD and catalase (Vasilaki *et al.*, 2006c; Palomero *et al.*, 2008).

Many adaptations to stress are attenuated in tissues from old mammals (Locke & Tanguay, 1996; Muramatsu *et al.*, 1996; Rao *et al.*, 1999; Hall *et al.*, 2000; Heydari *et al.*, 2000; Njemini *et al.*, 2002; Demirel *et al.*, 2003), and previous work from our group has shown that the adaptive responses of skeletal muscle to contraction-induced ROS are severely attenuated in aging through a lack of activation of transcription factors following contractile activity (Vasilaki *et al.*, 2006c). The mechanism for this attenuation of responses is unknown, but we hypothesize that the chronic ROS generation and adaptations that have occurred in the muscles of old mice prevent or mask the activation of the ROS signal. Further evidence for a role of a lack of signal for activation of the adaptive responses being responsible for failed adaptive responses in the muscles of old mice comes from work using the HSF activator, 17AAG, whereby treatment of mice with 17AAG resulted in the activation of HSF in the muscles of old mice (Kayani *et al.*, 2008a). 17AAG activates HSF by dissociating the transcription factor from one of its inhibitors, HSP90, and this study clearly demonstrated that bypassing the need for a signal for activation allows the activation process to function normally in the muscles of old mice. In the current work, we have examined whether a dysregulation in superoxide handling induced by a lack of CuZnSOD leads to a chronic increased generation of ROS and a failure of adaptive responses in the muscles of adult mice similar to that which occurs in old WT mice.

The data clearly show that muscles from adult *Sod1*^*−/−*^ mice have increased intracellular ROS activities at rest associated with constitutive activation of NFκB and AP-1 in muscle fibres (Figs 2D, 3A and 4) that are associated with an increased expression of HSP25 and HSP60 (Fig. 1C, D). HSP25 and HSP60 are potential products arising from the activation of these transcription factors, and previous data have shown that HSP25 content is elevated in the muscles of old compared with adult mice (Vasilaki *et al.*, 2006c). HSP60 is primarily a mitochondrial chaperone and so increased content may reflect an increase in mitochondrial number in the muscle as previously reported (Kostrominova *et al.*, 2007) although the increase in HSP60 content was 2.5-fold compared with the mitochondrial SOD2 which was only elevated by ∼ 40%. The period of contractile activity that induces an increase in ROS activities and stimulates adaptive responses in skeletal muscles from adult WT mice did not further increase ROS activities or activate adaptive responses in the muscles from adult *Sod1*^*−/−*^ mice (Fig. 5). The blunted adaptive responses in the muscles of the adult *Sod1*^*−/−*^ mice mimic those seen in the skeletal muscles of old WT mice (Vasilaki *et al.*, 2006c).

The *Sod1*^*−/−*^ mice appear to provide a useful model for investigating potential mechanisms by which increased superoxide may lead to loss of muscle mass and function during aging. The adult mice studied here showed an approximate 30% lower *gastrocnemius* muscle mass compared with age-matched WT mice (Table 1). No significant difference in body weight was seen in these mice and so this difference in muscle mass remains when expressed per unit body mass, demonstrating sarcopenia in these muscles. Previous data from these mice have shown that this decreased muscle mass becomes exacerbated with increasing age of the *Sod1*^*−/−*^ mice and the *gastocnemius* muscle is one of the first to demonstrate sarcopenia (Muller *et al.*, 2006). Data presented here demonstrate an association between the presence of sarcopenia and a failed adaptive response to contractions. This sarcopenia is associated with an increase in the markers of oxidative damage in muscle and other tissues (Elchuri *et al.*, 2005; Muller *et al.*, 2006) and by abnormalities in muscle fibre structure and innervation (Kostrominova *et al.*, 2007), all of which are normally found in old WT mice.

The present study has used two different approaches to evaluate whether the adult *Sod1*^*−/−*^ mice have an increase in skeletal muscle ROS activities in comparison with WT mice: the examination of interstitial ROS activities using microdialysis techniques (Vasilaki *et al.*, 2006a; Close & Jackson, 2008) and the use of DCFH as a nonspecific probe for intracellular ROS (McArdle *et al.*, 2005; Palomero *et al.*, 2008). The latter technique also required the isolation of intact single fibres from the FDB muscle to ensure that ROS activities were only measured from muscle fibres and eliminate interference from endothelial cells, white cells etc that can be a major source for ROS generation within the muscle bulk (Gomez-Cabrera *et al.*, 2010). Although it seemed intuitive that the muscle from adult *Sod1*^*−/−*^ mice would have increased ROS activities because of the lack of a major regulatory enzyme, the studies of interstitial ROS showed no difference in superoxide, NO or hydrogen peroxide in quiescent muscle from adult *Sod1*^*−/−*^ mice compared with quiescent muscle from WT mice. Intracellular DCFH oxidation was significantly higher in isolated fibres from adult *Sod1*^*−/−*^ mice compared with adult WT mice (Fig. 2D). Thus, these data are compatible with the possibility that an elevated intracellular ROS activity occurs in the muscle of adult *Sod1*^*−/−*^ mice at rest leading to a chronic activation of ROS-sensitive transcription factors.

During contractile activity, there was an increase in the interstitial superoxide activity as previously reported (McArdle *et al.*, 2001; Vasilaki *et al.*, 2006a), but with no difference between the muscles of adult *Sod1*^*−/−*^ and WT mice (Fig. 2). There was also a tendency for an increase in the hydrogen peroxide content in the interstitial fluid of muscle of the WT mice only. The DCF analyses indicated that there was a contraction-induced increase in intracellular oxidation in the fibres from WT mice, but this was not seen in the fibres from *Sod1*^*−/−*^ mice (Fig. 5B). These data indicate a failure of induction of any additional ROS by contractions in the muscles of *Sod1*^*−/−*^ mice. Muscle fibres were visibly contracting during these measurements although their force generation was not determined. Thus, these results are compatible with the hypothesis that the failure to induce adaptations to contractions in muscle of the *Sod1*^*−/−*^ mice is because of a failure to increase ROS activity in response to the contractile activity. It is inherently surprising that a lack of CuZnSOD does not lead to an inevitable increase in ROS activities in all situations, but no increase was seen following isometric contractions in comparison with WT mice. Muller *et al.* (2007a) report that *Sod1*^*−/−*^ mice show increased hydrogen peroxide release from mitochondria, and we hypothesize that this leads to the increased DCF formation in muscle fibres at rest that was seen here (Fig. 2D). The intracellular source(s) of the ROS leading to increased DCFH oxidation during contractions is not fully understood but may include nonmitochondrial sources for ROS generation (McArdle *et al.*, 2005; Vasilaki *et al.*, 2006a; Palomero *et al.*, 2008). Clearly, the modification in ROS activities in the cytosol is not reflected in the extracellular detection of ROS in this model. The effect of the lack of Cu,ZnSOD in *Sod1*^*−/−*^ mice on the nonmitochondrial sources of ROS generation, and the relationship between intracellular and extracellular ROS is the subject of current investigation.

In summary, these studies have demonstrated further similarities between the muscles of adult *Sod1*^*−/−*^ mice and those of old WT mice. The *Sod1*^*−/−*^ mice show an attenuated ability to respond to the stress of contractile activity. Studies using transgenic mice overexpressing HSPs have demonstrated that a lack of ability to increase the expression of HSPs following contractions plays an important role in age-related loss of skeletal muscle mass and function (McArdle *et al.*, 2004a; Broome *et al.*, 2006; Kayani *et al.*, 2008a, b), and we speculate this may also play a role in the accelerated age-related loss of muscle mass seen in the *Sod1*^*−/−*^ mice. The data also imply that a chronic increase in ROS activities caused by a lack of Cu,ZnSOD can lead to this failure of adaptation to contractions, although the specific ROS species involved and their intracellular sources have not been identified.

## Experimental procedures

### Mice

Adult male WT C57Bl6 mice and adult (6–8 months) male littermate *Sod1*^*−/−*^ mice were used in this study. All mice were male. Mice were generated as previously reported (Muller *et al.*, 2006; Kostrominova *et al.*, 2007) and fed on a standard laboratory diet and subjected to a 12-h light/dark cycle. Experiments were performed in accordance with UK Home Office guidelines under the UK Animals (Scientific Procedures) Act 1986 and received ethical approval from the University of Liverpool Animal Welfare Committee.

### *In vivo* isometric contraction protocol

Adult WT and *Sod1*^*−/−*^ mice were anesthetized with pentobarbital sodium, with an initial dose of 65 mg per 100 g of body mass via an intraperitoneal injection. Supplemental doses were administered as required to maintain a depth of anaesthesia sufficient to prevent response to tactile stimuli. The knee of one hind limb was fixed to a base plate, and the hindlimb musculature was stimulated to contract by surface electrodes placed around the upper limb and the ankle to induce isometric contractions under *in vivo* conditions (McArdle *et al.*, 2001). Fibre length was set at the optimum length for force production. Maximum isometric tetanic contractions were produced by square wave pulses of 0.2-ms duration, a voltage slightly greater than that required to produce a maximum twitch (usually ∼ 70 V), and a frequency of 100 Hz (Brooks & Faulkner, 1991; McArdle *et al.*, 2001). Maximum isometric contractions were held for 500 ms, with a contraction every 4 s for a total of 225 contractions during the 15 min of the contraction protocol. Mice remained under anaesthesia until the end of the contraction protocol. Mice were allowed to recover and killed by cervical dislocation at 15 min following the end of the contraction protocol. Mice were then weighed, and g*astrocnemius* muscles were removed, weighed and frozen in liquid nitrogen.

For muscle samples from quiescent mice, mice were euthanized by overdose of anaesthesia and the gastrocnemius muscles were removed for biochemical analyses without undergoing the contraction protocol. These muscles were rapidly frozen in liquid nitrogen and stored at −70°C until analysed.

### Measurement of interstitial ROS by *in vivo* microdialysis

The superoxide anion content, hydrogen peroxide content and nitrate and nitrite content in the interstitial fluid from quiescent muscles were obtained by sampling the extracellular space with microdialysis probes placed in the GTN muscles (McArdle *et al.*, 2001; Vasilaki *et al.*, 2006a). Three microdialysis probes (MAB 3.8.4; Metalant AB, Stockholm, Sweden) with a molecular weight cut-off of 35 000 Da were placed into the *gastrocnemius* muscles of both limbs of anesthetized animals using a 22G plastic introducer. The probes were perfused with either normal saline (for the analysis of hydrogen peroxide and nitrate and nitrite) or 50 μm cytochrome *c* in normal saline (for analysis of superoxide anion) at a flow rate of 4 μL min^−1^ and allowed to stabilize for 60 min. Samples were collected from the probes over sequential 15-min periods prior to the contraction protocol, during the protocol and for 15 min following the contraction protocol.

Reduction of cytochrome *c* in the microdialysate was used as an index of superoxide anion concentration in microdialysates (McArdle *et al.*, 2001). The total nitrate and nitrite content of microdialysates was measured as an index of total NO generation using a commercial fluorometric assay (Cayman Chemical Co., Ann Arbor, MI, USA) based on the method of Miles *et al.* (1995). The hydrogen peroxide content of microdialysates was measured using a modification of the method of Lei *et al.* (1998).

### Determination of NFkB and AP-1 DNA binding activity in gastrocnemius muscles

Transcription factor DNA binding activity of whole cell or nuclear extracts from skeletal muscles was measured by EMSA as previously described (Broome *et al.*, 2006; Vasilaki *et al.*, 2006c). Protein extracts were prepared according to the method described by Mosser *et al.* (1988). For DNA binding reactions of NF-κB oligonucleotide and AP-1 oligonucleotide (listed below; Promega UK, Southampton, UK), 100 μg of extract was mixed with 16 fmol of either NF-κB-or AP-1-radiolabelled oligonucleotides in binding buffer (Prepared from 5 × binding buffer containing 50 mm Tris–HCl (pH 7.5), 2.5 mm EDTA, 20% (v/v) glycerol, 2.5 mm DTT, 0.25 mg mL^−1^ poly(dI-dC)•poly(dI-dC), 5 mm MgCl_2_; Promega Corporation, Madison, WI, USA) to a final volume of 20 μL. The reactions were incubated at room temperature for 20 min. Following incubation, samples were loaded onto a 4% polyacrylamide gel (National Diagnostics Ltd, East Riding, UK) in electrophoresis buffer. Electrophoresis was carried out at room temperature for 1 h at 350 V using a Biorad DCode™ System (Biorad, Hercules, CA, USA). Gels were exposed to a phosphor screen overnight (Amersham International, Buckinghamshire, UK). The phosphor screen was scanned using Biorad Personal Molecular Imager FX (Biorad) and analysed using Quantity One Software (Biorad).

NF-κB oligonucleotide: 5′-AGTTGAGGGGACTTT-CCCAGGC-3′

AP-1 oligonucleotide: 5′-CGCTTGATGAGTCAGCCGGAA-3′

To verify that the results from EMSA analysis did not arise from nonspecific binding, competition experiments and supershift assays were also performed as previously described (Vasilaki *et al.*, 2006c). Electrophoretic mobility shift assays were quantified by densitometry, and DNA binding activities were expressed relative to the DNA binding activity of WT control samples on the same gel.

For immunolocalization of p65, 10-μm transverse sections were cut from *gastrocnemius* muscle, fixed in 4% paraformaldehyde in phosphate-buffered saline (PBS) and subsequently blocked in 1% BSA in PBS for 30 min. This was followed by incubation in primary (rabbit anti-mouse NFκB; Abcam Inc, Cambridge, MA, USA) and secondary (IgG conjugated with Alexa fluor 405; Molecular Probes, Invitrogen, Eugene, OR, USA) antibodies. All nuclei were stained using propidium iodide 1:1000 dilution (Invitrogen, Eugene, OR, USA). Negative control slides were used to ensure the specificity of p65 staining, and slides were mounted in Dako Fluorescent Mounting Medium (Dako, Glostrup, Denmark A/S). Sections were imaged using a Nikon E-Ti inverted microscope with a C1 confocal (Nikon Instruments Europe BV, Surrey, UK) comprising a diode (UV) 405-nm excitation, an argon laser 488-nm excitation and a helium-neon with 543-nm excitation. Acquisition software was EZC1 V.3.9 (12 bit). Anti-rabbit secondary antibodies were excited using a 405-nm diode laser passing through a main dichroic and secondary beam splitter with the emission collected through a 450/35 filter to a detector. Propidium iodide was excited using 543-nm laser passing through a main dichroic and secondary beam splitter with the emission collected through a 605/75 filter to a detector.

### Determination of total glutathione and protein thiol content of muscles

The automated glutathione recycling method described by Anderson (1985) was used to assess the total glutathione content of muscle samples, using a 96-well plate reader (Benchmark; Bio-Rad, Hemel Hempstead, UK). The protein thiol content of samples was analysed by the method of Di Monte *et al.* (1984) adapted for use on a 96-well plate reader.

### Determination of the content of HSPs and antioxidant defence proteins in muscles

For the assessment of protein content of HSPs, 100 μg of total protein was applied to a 12% polyacrylamide gel with a 4% stacking gel (National Diagnostics Ltd). The separated proteins were transferred onto nitrocellulose membranes by western blotting. The membranes were analysed using antibodies to HSP60 (SPA-815; Stressgen Inc., Victoria, British Columbia, Canada), HSP25 (SPA-801; Stressgen Inc.), MnSOD (SOD-110; Stressgen Inc.) and CuZnSOD (SOD-101; Stressgen Inc.) as previously described (McArdle *et al.*, 2001). The membranes were incubated with anti-mouse or anti-rabbit peroxidase-conjugated IgG antibodies where appropriate (Sigma Co., Dorset, UK, A2554 or A0545, respectively). Peroxidase activity was detected using an enhanced chemiluminescence kit (Amersham International, RPN3004), and bands were visualized as described previously (McArdle *et al.*, 2001). All western blots were carried out a minimum of three times, and bands were identified in comparison with a sample that had not been exposed to the primary antibody and using molecular weight markers (Amersham International). To allow comparison of the absolute levels of HSPs in the muscles of WT and genetically altered mice, the content of each HSP is expressed as a percentage of the content of adult WT quiescent muscle as described in the figure legends.

### Measurement of intracellular ROS activities by monitoring DCFH oxidation in single isolated muscle fibres

Following sacrifice of WT or *Sod1*^*−/−*^ mice that did not undergo the *in vivo* contraction protocol, the FDB muscles were removed and placed into 0.4% type I collagenase (EC 3.4.24.3) (Sigma–Aldrich Co., St Louis, MO, USA) solution in culture medium. This was composed of minimum essential medium Eagle (MEM) (Sigma–Aldrich Co.) supplemented with 10% foetal bovine serum (FBS) (Invitrogen Ltd, Paisley, UK) containing 2 mm glutamine, 50 i.u. penicillin and 50 μg mL^−1^ streptomycin. Both FDB muscles from each mouse were incubated in collagenase solution at 37°C for 2 h, and the mixture was manually shaken every 30 min to improve digestion of the connective tissue. Fibre bundles that had not been separated during the incubation were gently triturated by a wide-bore plastic pipette to separate the fibres. Free single muscle fibres were separated from damaged fibres and contaminating cells by centrifugation at low speed (600 *g* for 30 s). The fibres were washed four times in fresh culture medium. Cleaned fibres were plated onto 35-mm dishes precoated with 40 μL of Matrigel (BD Biosciences, Sparks, MD, USA). Fibres were cultured for 24 h at 37°C in a 5% CO_2_ atmosphere (Pye *et al.*, 2007; Palomero *et al.*, 2008).

A solution of 5- (and 6-) chloromethyl-2′,7′-dichlorodihydrofluorescein diacetate (CM-DCFH DA) (Molecular Probes™, Invitrogen, Eugene, OR) was prepared daily in absolute ethanol and kept at 4°C. Culture plates containing isolated fibres were washed with Dulbecco’s phosphate-buffered saline (D-PBS), (Sigma-Aldrich Co.), and fibres were pre-incubated in D-PBS at 37°C for 30 min. The medium was then replaced by D-PBS containing CM-DCFH DA (17.5 μm) and incubated for 30 min at 37°C. The fibres were then washed with D-PBS and covered with MEM without Phenol Red (Sigma-Aldrich Co.) for fluorescence microscopy.

The imaging system consisted of a Zeiss Axiovert 200 M epifluorescence microscope equipped with ×10 and ×20 objectives and filter sets: a 450- to 490-nm excitation/515- to 565-nm emission filter set that was used for CM-DCF fluorescence and a 365-nm excitation/420-nm emission filter set that was used for 4′,6-diamidino-2-phenylindole (DAPI) fluorescence (Carl Zeiss Microimaging GmbH, Jena, Germany). Images were acquired and analysed using a computer-controlled Zeiss HRc charged-coupled device camera (Carl Zeiss Microimaging GmbH) and by AxioVision 4.4 image capture and analysis software (Carl Zeiss Microimaging GmbH) for quantification of changes in emission fluorescence. This software allows measurements to be made from user-defined areas of the microscope field; in this case, the fluorescence measurements were localized to selected areas of the muscle fibres.

CM-DCF fluorescence from fibres was recorded at 15-min intervals over 45 min at 25°C. Exposure of fibres to ultraviolet (UV) light was minimized by use of a 500-ms exposure time.

Isolated fibres were stimulated to contract, as previously described (Pye *et al.*, 2007; Palomero *et al.*, 2008). In brief, platinum electrodes were placed into the well and provided trains of biphasic square wave pulses of 2 ms in duration for 0.5 s repeated every 5 s at 50 Hz and 30 V/well. The total stimulation time was 15 min, and this commenced at 15 min following the beginning of the experiment.

### Statistics

Statistical analysis was carried out using Statistical Package for Social Sciences (SPSS, Feltham, Middlesex, UK) software version 15. Where multiple comparisons were made, data were analysed using anova. When a significant *F* value was observed, least significant difference *post hoc* analysis was performed to identify where differences occurred. Data are presented as means ± SE for each experiment, and data were considered significant at *P* < 0.05.
